# Stigma of Maize and Avena Sativa

**Published:** 1881-10

**Authors:** W. G. Owen

**Affiliations:** Atlanta, Ga.; Professor of the Principles and Practice of Medicine in the Southern Medical College, Atlanta


### STIGMA OF MAIZE AND AVENA SATIVA.

By W. G OWEN, M.D., Atlanta, Ga.
Professor of the Principles and Practice of Medicine in the Southern Medical College, Atlanta.

Several times during the past year I have noticed allusions to the virtues of stigma of maize (fluid extract of corn silk) in the treatment of cystitis and other affections of the bladder and genito-urinary organs, and avena sativa (concentrated tincture of common oats) in the treatment of various disorders of the nervous system. As they are comparatively new remedies and seem not to have attracted much attention, I presume to testify to their merits from personal observation, with the hope of aiding in their speedy introduction into general use. I have only time at present to say that I have used the maize in three cases of cystitis. One resulting from the vesical irritation arising from subinvolution of the womb. The desire to pass water was almost continual—but a small quantity voided each time and always with intense burning pain. It was highly colored (claret), acid and loaded with mucus and epithelial scales. The other two were similar in character but from different causes. The usual remedies in such cases having failed, I resorted to the maize in drachm doses every four hours. In the first two decided improvement was manifest after the third dose. The intervals between the paroxysms longer, the quantity of urine increased, and the pain mitigated. At the end of the second day the urine was nearly normal, the pain subdued and the patients satisfied. As a precaution against relapse while the original cause remained, I continued the drachm doses every four hours for one week. Two months have elapsed and no return. The other case failed to yield until I gave drachm doses every two hours. After twenty-four hours I had the satisfaction of seeing my patient relieved. I have not attempted to describe these cases at length, nor to give the analysis at different periods of the attacks, nor have I considered it at all necessary to enter into a long treatise on the causes, duration or the pathology of cystitis. My only object is to call the attention of the profession to what I believe to be a valuable remedy in this class of cases. The preparation I used was prepared by Lamar, Rankin & Lamar of this city.

The concentrated tincture of avena sativa I regard invaluable as a nerve stimulant and tonic. Ergot has entered conspicuously into our neuro-therapeutics, and a valuable remedy it is, but there are many cases in which we dare not employ it. The avena possesses all its merits and the additional one of being admisible in cases where the pregnant state would bar the use of the ergot. I have used it in epilepsy, chorea, constipation, diminished peripheral nerve action, sleeplessness and a variety of disorders depending upon a neurasthenic condition. I do not believe in specifics, nor do I regard these remedies as entirely worthy of confidence from my limited experience in their use, but so far, I can safely say that they have exceeded my expectations and I greatly regret that I cannot now enter more fully into the detail of each case treated. The dose of avena is ten to thirty drops three or four times a day. The preparation used was prepared by B. Keith & Co. New York.

